# Hafted technologies likely reduced stone tool-related selective pressures acting on the hominin hand

**DOI:** 10.1038/s41598-023-42096-z

**Published:** 2023-09-20

**Authors:** Anna Mika, Julie Lierenz, Andrew Smith, Briggs Buchanan, Robert S. Walker, Metin I. Eren, Michelle R. Bebber, Alastair Key

**Affiliations:** 1 Department of Archaeology, University of Cambridge, Cambridge, CB2 3DZ UK; 2 Department of Anthropology, Kent State University, Kent, OH 44224 USA; 3 Department of Anthropology, Ohio State University, Columbus, OH 43210 USA; 4 Department of Anthropology, University of Tulsa, Tulsa, OK 74104 USA; 5 Department of Anthropology, University of Missouri, Columbia, 65211 USA; 6 Department of Archaeology, Cleveland Museum of Natural History, Cleveland, OH 44106 USA

**Keywords:** Archaeology, Cultural evolution

## Abstract

The evolution of the hominin hand has been widely linked to the use and production of flaked stone tool technologies. After the earliest handheld flake tools emerged, shifts in hominin hand anatomy allowing for greater force during precision gripping and ease when manipulating objects in-hand are observed in the fossil record. Previous research has demonstrated how biometric traits, such as hand and digit lengths and precision grip strength, impact functional performance and ergonomic relationships when using flake and core technologies. These studies are consistent with the idea that evolutionary selective pressures would have favoured individuals better able to efficiently and effectively produce and use flaked stone tools. After the advent of composite technologies during the Middle Stone Age and Middle Palaeolithic, fossil evidence reveals differences in hand anatomy between populations, but there is minimal evidence for an increase in precision gripping capabilities. Furthermore, there is little research investigating the selective pressures, if any, impacting manual anatomy after the introduction of hafted composite stone technologies (‘handles’). Here we investigated the possible influence of tool-user biometric variation on the functional performance of 420 hafted Clovis knife replicas. Our results suggest there to be no statistical relationships between biometric variables and cutting performance. Therefore, we argue that the advent of hafted stone technologies may have acted as a ‘performance equaliser’ within populations and removed (or reduced) selective pressures favouring forceful precision gripping capabilities, which in turn could have increased the relative importance of cultural evolutionary selective pressures in the determination of a stone tool’s performance.

## Introduction

### Co-Evolution of the human hand and stone tool technology

The emergence of flaked stone tool technologies has been linked with major shifts in the evolution of the hominin hand. From Darwin^1^ onwards, there is considerable discussion of how the dexterous and manipulatively forceful human (*Homo sapiens*) hand evolved relative to the selective pressures experienced by other extant primates. The intentional production and use of flaked stone tools, one of the few behavioural traits unique to hominins during the Plio-Pleistocene^2–4^, provides one of the clearest routes to explaining such differences.

Our hand is functionally derived in unique ways compared to other extant primate species. Humans possess a long and robust thumb (1st digit) relative to the length of the other manual digits, and while the uniqueness of this trait is debated^5,6^, it nonetheless facilitates our unrivalled dexterity and ease of opposition between the distal phalanges^7–10^. We also have a fully formed flexor pollicus longus muscle, which aids forceful flexion of the first distal phalanx during precision gripping^11–14^. Further, humans possess large volar pads and apical tufts, which facilitate the distribution of high forces over a large surface area^15^. Other features of our manual anatomy, including carpometacarpal and metacarpophalangeal joint morphologies, the third metacarpal styloid process, and multiple aspects of our muscular architecture, also contribute to our ability to exert and resist substantial manual forces, or effectively manipulate objects, in a way that is not evidenced in other primates^8,9,14^.

To better understand the influence of stone tool-related behaviours on the evolution of the hominin hand, diverse experiments have investigated how the human hand interacts with stone tools during their transportation, manufacture, and use^11,12,16–25^. We direct readers to Marzke^8^ and Key and Lycett^26^ for reviews of these experiments. Among other results, such studies confirm the thumb to be highly and heavily recruited during both stone tool use and production, the index finger (2nd digit) to be essential to the effective use of flake and core tools, and a few key precision grips to characterise most stone tool use activities.

While the aforementioned studies are important for demonstrating how our unique manual anatomy may be evolutionarily linked with flaked lithic technologies, few directly investigate the strength of any proposed selective pressures. That is, most studies investigate the comparative recruitment of individual anatomical elements during stone tool behaviours (e.g., digit one relative to digits two to four) and link derived anatomy to any variation observed. Few studies investigate how anatomical variation at a population level influences one’s ability to effectively or efficiently produce or use flaked stone technologies. This distinction is important, as the latter can provide direct evidence in support of the selective pressures underpinning any hypothesised evolutionary links determined by the former. For example, if our relatively long and robust thumbs evolved in response to hammerstone use^23^ (among other activities^8,9,22^), then individuals displaying variation in the expression of these traits should experience varying capacities to secure hammerstones during their use, alongside different levels of joint damage and joint/muscular discomfort, the capacity to exert greater or lesser gripping forces, and varying fatiguing rates^18^. Indeed, if such costs and benefits are argued to underpin the evolution of hominin manual anatomy in the Early Stone Age (ESA), then *Homo sapiens* population-level variation in these traits should similarly result in differences enough to hypothetically induce evolutionary change.

Only a few studies^18,22,27–29^ have actively demonstrated how biomechanical and/or biometric variation in the human upper limb influences the efficacy of stone tool-related behaviours. This includes Key and Lycett^27^ who demonstrated flake cutting efficiency to be significantly related to tool user hand size (which is itself highly correlated with grip strength^30^). Rolian et al.^18^ demonstrated increased digit length to correlate with a reduction in joint contact stress and muscle force during hammerstone and flake tool use. Fedato et al.^29^ investigated haptic responses to handaxe and flake tool handling in *Homo sapiens*, revealing hand morphology to have little to no relationship with electrodermal activity. Key and Lycett^22,28^ provide the only other studies to investigate shaped core technologies, demonstrating there to be significant positive relationships between digit lengths, digit ratios and gripping strength (pinch and power) and handaxe cutting and loading performance; this in turn has implications for other large non-hafted cutting tools. Several studies have modelled the varying manual capabilities of hominins based on the 3D morphology of fossil carpals, metacarpals and phalanges^31–33^, but it is difficult to ascertain strength of relationships at a population level as sample sizes are, unavoidably, low. Moreover, while such studies are valuable and manual capabilities can be estimated, 3D modelling efforts do not demonstrate whether costs and/or benefits are realised in real-world conditions.

These studies only investigate a few Early Stone Age (ESA) and Lower Palaeolithic (LP) technologies, and although flakes and large cutting tool (LCT) bifaces characterise two to three million years of the archaeological record, there is little to no understanding of the selective pressures experienced by the hominin hand after the advent of the Middle Stone Age (MSA) and Middle Palaeolithic (MP) (although see ^33–37^). Simply, we have little evidence concerning the selective pressures experienced by the hominin hand after this point, despite there being major changes to how the hand interacts with these more complex, and often smaller^38^, tool types^26,39^.

### Determining the role of biometric variables on the performance of composite technologies

Perhaps the most important MSA/MP-associated technological development, at least in terms of hand-tool interactions, is the advent of hafted composite tool technology^39^. Prior to stone flakes, blades, points and bifaces being attached to a handle, stone tools were hand-held, with no barrier or structure between the tool-user’s hand and flaked stone object^39^. Earlier non-hafted tool types were secured in the hand using precision grips, principally by the forceful flexion of the first distal phalanx, the second digit, and their recruitment in opposition to each other and digits three to five^17,22,40^. With the introduction of hafting, there was a trend towards more complex technologies and increased effectiveness during cutting, scraping, chopping, and piercing actions^36,37,39,41^. Simultaneously, there was a shift from the recruitment of precision grips to power grips (see ^17,40^), and in turn, a change in the musculoskeletal demands placed on the upper limb^36,37,42^.

The handles of composite tools (including knives, which can be defined as hand-held composite tools formed from an elongated sharp-edge object and handle) are thought to increase a user’s control over the tool’s angle of application, cutting direction, and working forces. Handles are also hypothesised to increase the precision, manipulability, and manoeuvrability of the tool by eliciting greater flexibility and ranges of motion^39^. Further, handles are known to provide increased muscular comfort relative to earlier stone tools^36,37^. This was recently evidenced by both Key et al.^36^ and Coe et al.^37^, who demonstrated ergonomic differences between hafted and non-hafted tools. While non-hafted tools elicited muscle activation predominantly within the muscles of the forearm, it was shown that muscle activation of the shoulder was increased during hafted tool use. Moreover, muscle activation in the hand was significantly greater during the use of non-hafted tools. These studies support the efficiency hypothesis by demonstrating hafted tools to be functionally and ergonomically more efficient than non-hafted alternatives. Yet the impact that individual tool users can have on these performance characteristics (*c.f*.^43^) is unknown, and in turn, it is currently difficult to ascertain whether the selective pressures evidenced for earlier lithic technologies may have continued or been altered.

Hand-held hafted stone technologies vary widely in form throughout the Mid-to-Late Pleistocene and the question of why this variation exists has been the focus of numerous studies. North American Clovis points provide a useful case study as they have been subject to intense investigation, which has suggested their forms are likely a result of multiple factors, including cultural drift, non-functional bias, selection of functional features, as well as heritable and non-heritable sources of variation^44–63^. Form-function relationships are particularly important in the present context, as it has recently been demonstrated—at least for Acheulean handaxes—that the biometric attributes of tool-users have a greater impact on cutting performance than the morphology of the tool itself^28^. Thus, if the performance characteristics of hafted stone technologies are influenced by tool-user variation to a similar extent, then the manual selective pressures evidenced prior to the MSA and MP may continue after this point.

Here, we investigate the influence of tool-user biometric variation on the functional performance of 420 hafted Clovis point replicas that vary in their plan-view form. We test the hypothesis that if stone tool-use related selective pressures continued to act on hominin hand anatomy after the advent of composite hafted cutting tools, then we would expect to see statistically significant differences in tool performance between individuals based on their anatomy. If no statistical differences are observed, then it could be argued that the advent of hafted stone technologies (i.e., ‘handles’) acted as a ‘performance equaliser’ within populations. We focus on manual features linked to hominin precision gripping capabilities due to their strong evolutionary association with Palaeolithic technologies. An absence of biomechanically related performance differences between individuals (i.e., equalised performance) would suggest the removal or reduction of stone tool-related selective pressures favouring increased precision gripping capabilities during the MSA and MP. This in turn would have increased the relative importance of cultural evolutionary selective pressures (such as form-function relationships) in the determination of a stone tool’s performance.

## Results

**Figure 1:**
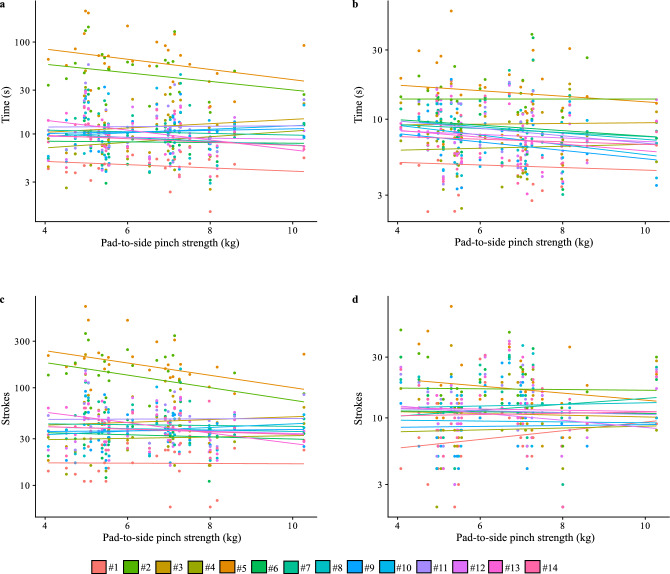
Bivariate plots of average pad-to-side pinch strength for the two tasks measured in time and number of strokes. Best-fit lines and individual observations are coloured by knife number. (**a**) pad-to-side pinch strength and time for task 1, (**b**) pad-to-side pinch strength and time for task 2, (**c**) pad-to-side pinch strength and stroke count for task 1, and (**d**) pad-to-side pinch strength and stroke count for task 2.

**Figure 2:**
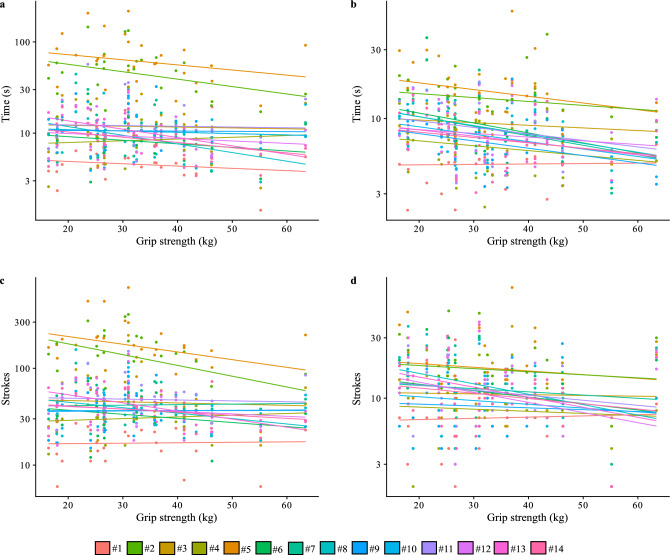
Bivariate plots of grip strength of dominant hand for the two tasks measured in time and number of strokes. Best-fit lines and individual observations are coloured by knife number. (**a**) grip strength and time for task 1, (**b**) grip strength and time for task 2, (**c**) grip strength and stroke count for task 1, and (**d**) grip strength and stroke count for task 2.

Figures 1, 2, and Supplementary Figures S1, S2, and S3 show that grip strength, pad-to-side pinch strength, digit ratio, thumb length, and hand length generally have little overall relationship with the number of cut strokes or time for both tasks. While two smaller points (point numbers 2 and 5) do show that more strength is associated with more efficient cutting, the overall effect is small and not significant.

For all four models of average pad-to-side pinch strength for the cutting tasks measured with stroke count or time the posterior distributions overlapped with zero suggesting that average pinch strength is not an important factor (Table 1). The estimates of the intercepts in these models shows that it takes on average more strokes and time to accomplish task 1, cutting through rope, than task 2, cutting through clay, when accounting for pinch strength.

**Table 1:** Bayesian regression model results for tests of average pad-to-side pinch strength for stroke count and time for task 1 and 2. The lower (L-95% CI) and upper (U-95% CI) credible intervals for the posterior distribution of slopes all overlap zero indicating non-significance.

	Estimate	Est. Error	L-95% CI	U-95% CI
Model 1: Pad-to-side pinch strength, stroke count for task 1				
Intercept	51.80	12.11	28.22	75.64
Pinch strength	− 0.05	0.49	− 1.02	0.90
Model 2: Pad-to-side pinch strength, stroke count for task 2				
Intercept	13.29	3.14	7.07	19.48
Pinch strength	− 0.02	0.43	− 0.88	0.83
Model 3: Pad-to-side pinch strength, time for task 1				
Intercept	15.29	5.32	4.70	25.73
Pinch strength	− 0.13	0.47	− 1.03	0.79
Model 4: Pad-to-side pinch strength, time for task 2				
Intercept	10.20	2.41	5.37	14.84
Pinch strength	− 0.13	0.33	− 0.79	0.53

Similarly, the four models of grip strength of the dominant hand for the cutting tasks measured with stroke count or time also had posterior distributions that overlapped zero suggesting that grip strength is not an important factor (Table 2). The estimates of the intercepts in these models shows that it takes on average more strokes and time to accomplish task 1, cutting through rope, than task 2, cutting through clay, when accounting for grip strength.

**Table 2:** Bayesian regression model results for tests of grip strength of dominant hand for stroke count and time for task 1 and 2. The lower (L-95% CI) and upper (U-95% CI) credible intervals for the posterior distribution of slopes all overlap zero indicating non-significance.

	Estimate	Est. Error	L-95% CI	U-95% CI
Model 1: Grip strength, stroke count for task 1				
Intercept	61.93	18.18	26.03	97.95
Grip strength	− 0.26	0.36	− 0.95	0.45
Model 2: Grip strength, stroke count for task 2				
Intercept	16.13	3.73	8.77	23.48
Grip strength	− 0.09	0.11	− 0.30	0.12
Model 3: Grip strength, time for task 1				
Intercept	20.42	7.60	5.76	35.60
Grip strength	− 0.16	0.16	− 0.46	0.15
Model 4: Grip strength, time for task 2				
Intercept	12.13	2.13	8.00	16.36
Grip strength	− 0.09	0.06	− 0.20	0.03

## Discussion

Diverse aspects of hominin manual anatomy may have been under selective pressure as a result of using and producing flaked lithic technologies. Stone tools are hypothesised to have been important to the survival of Lower Palaeolithic and Early Stone Age hominins^64,65^—albeit potentially progressively so^66–68^—and in turn, those individuals with anatomy better able to efficiently and effectively produce and use these technologies may have had an adaptive advantage. In support of this hypothesised evolutionary link, previous research has experimentally shown how modern *Homo sapiens* biometric variation in the hand significantly influences the efficacy of stone tool use behaviours^16,18,27,28^, and how during stone tool use, derived features in the hand can be highly recruited^8,11,17,23,69^. The impact of hafting (i.e., the development of handles) on the proposed evolutionary relationships between stone tool-use efficiency and hominin hand evolution has not, however, been experimentally investigated.

Presented here are data investigating the influence of *Homo sapiens* manual biometric variation on the functional performance of hafted (replica) Clovis points. Our results returned no statistically significant relationships between precision pad-to-side pinch strength, grip strength, digit ratio, thumb length, or hand length (Table 1 and 2; Supplementary Tables S1–S3) and the cutting efficiency of these hafted stone points. Accordingly, contrary to our expectations and the relationships previously established for directly hand-held flake and handaxe technologies, manual biometric variation does not appear to significantly increase the efficiency with which hafted knife technologies can be used. The advent of hafted composite stone technology during MSA/MP could, therefore, provide evidence of a ‘technological equaliser’ among hominin populations, whereby individuals with an arguably less advantageous anatomical condition in their upper limb (e.g., lower precision pinch strength potential) could have used stone tool technologies with comparable efficiency to individuals with more favourable anatomy. In turn, this could have removed evolutionary selective pressures associated with the use of flaked stone tool technologies; particularly those favouring increased precision pinch capabilities in hominins. This is not to say that unhafted technologies disappeared and were completely removed from the Palaeolithic toolkit upon the advent of hafted technologies. Nor that selective pressures were completely removed in all populations. Rather, manual selective pressures derived from the use of unhafted tools would have been proportionally reduced in populations relative to the proportional increase in hafted tools versus non-hafted.

### Hominin hand evolution in light of these experimental data

Hominins would likely have been capable of precision gripping, potentially with a degree of force, prior to the advent of flaked stone technologies. Morphometric and modelling-based studies of early hominin fossil anatomy, and investigations of *Pan* manual capabilities, supports this inference. For example, *Pan* is known to use precision grips to secure tools and food items with a degree of force^70–72^, while the digit ratios of *Australopithecus afarensis* indicate that they may have been capable of securing flakes between their thumb and index finger^73,74^ (although see ^5^). Further, studies are increasingly demonstrating that some australopithecine species did perform precision grips with force and frequency enough to remodel the trabecular structure of their metacarpals^75,76^. Although, notably, there is temporal overlap between the hominin species in these latter studies and early stone tool occurrences.

Subsequent to the advent of Oldowan (and potentially ‘Lomekwian’, but see ^77,78^) technologies between 3.3 and 2.8 ma^3,79,80^, hominins are suggested to have evolved increased precision gripping capabilities as a result of stone tool-related selective pressures. Indeed, multiple derived musculoskeletal structures in the hand are adaptively linked to the use of flake technologies (see introduction^8,9,17^). The shared derived features of *Australopithecus* and *Homo* manual anatomy, relative to *Ardipithecus*, may even suggest stone tool related selective pressures favouring increased forceful precision griping capabilities to have been present in our lineage prior to c. 3.3–2.8 ma^81^. Our data suggest that the advent of hafted technologies may have reduced or removed those selective pressures in later hominin populations.

Unfortunately, the early *Homo* fossil record displays few manual elements, with the famous OH7 remains being the principal evidence for *Homo habilis*, and a 1.4 ma third metacarpal (West Turkana, Kenya) and 1.2–1.3 ma proximal phalanx (Atapuerca, Spain) providing some of the few pieces of evidence in hand for *Homo erectus s.l.*^82–84^. Although the evolution of the early *Homo* hand is thought to have undergone evolutionary change during early Pleistocene^8,84–86^, the transition to, and anatomical condition in, the *H. erectus* hand remains unresolved. Unfortunately, this coincides with the earliest suggested emergence of hafted technologies^39^. By the time that we have more secure evidence of hafting during the late Middle Pleistocene^39,87–89^, *Homo* is inferred to display manual anatomy capable of *H. sapiens*-like forceful precision gripping abilities ^31,86,90,91^. Thus, while our data suggests hafting to have potentially limited further changes to the precision gripping capabilities of hominins, it is currently difficult to identify when these selective pressures may have been lifted using either fossil or archaeological evidence.

This does not mean hafted technologies did not exert their own distinct evolutionary pressures on the hominin hand. Indeed, Neanderthals have been suggested to be more heavily adapted for power gripping than *Homo sapiens*, which may possibly be related to the use of Middle Palaeolithic toolkits, including their hafted components^33,34,92^. This suite of “subtle morphological differences between the [trapeziometacarpal] complexes of Neanderthals and modern humans”[33:2] includes a large and dorsopalmarly flatter trapezial-MC1 joint, reduced 3rd metacarpal styloid process, radioulnarly flat fifth metacarpal bases and parasagittally-oriented capitate-second metacarpal facets^33,92^. Although there is a lack of clarity regarding the role of developmental and genetic processes in these differences and Neanderthals still display evidence for habitually performing forceful precision grips^75,86^. Further, recent electromyography data^36,37^ and long-standing grip data^39,42,93^ link hafted stone tools with reduced muscular demands in the hand relative to non-hafted alternatives. Potentially, it could even be the case that the more robust phalanges, and broader distal phalanges, observed in Neanderthals relative to *H. sapiens* are linked to the former’s greater reliance on unhafted tools (and in turn the continuation of selective pressure acting on precision gripping capabilities^86^). Untangling how the reduced musculoskeletal demands of hafted technologies influenced Neanderthal hand anatomy given these populations continued to use unhafted, precision held^16,21,69^, stone and organic technologies remain unresolved.

It is notable that the typically *Homo-*like styloid process on the third metacarpal, which aides in “stabilising the [third carpometacarpal joint] joint against mechanical loads generated during power grips when making and using tools” (p. 628)^94^, is present in *H. erectus* by at least 1.42 ma^83^, and that this occurred after the onset of the Acheulean but before the advent of hafted technologies^39^. The absence of the third metacarpal styloid process in hominins contemporary to the Oldowan is important. Not only does it suggest that these typically small (3–5 cm) flake technologies did not require the associated increased metacarpal-carpal stabilisation, but it reveals that the *use* of Acheulean handaxes and cleavers may have provided the necessary selective pressures to evolve this anatomical feature. The broad pad-to-pad and pad-to-side grips associated with handaxe and cleaver use^22,24,95^, and their increased muscular force requirements and working force capabilities relative to Oldowan flakes^69^, support this inference. The use of non-stone tipped spear technologies in the early Acheulean could have created similar selective pressures associated with the use of power grips^96^, but whether such technologies were habitually used by early *H. erectus* remains unknown.

### Notes on cutting technique, fatigue and task design

While this study did not investigate the impact of fatiguing rates on tool performance, some relevant observations were made. Despite breaks being given between each cutting trial, participants occasionally commented that they experienced a degree of fatigue *during* the cutting tasks. Most notably during the use of Knife #2–Shoop #1 and Knife #5–Shoop #2. These knife forms consisted of the smallest blades, with the blade lengths ranging from 30.21 to 36.85 mm (Shoop #1) and 29.95–35.97 mm (Shoop #2). When considering that the blades were also hafted, this further reduced the amount of available cutting edge. Thus, it is not surprising that these short-bladed knives elicited fatiguing. This is consistent with Mika et al.^62^, where the two smallest knife forms, Shoop #1 and #2, were identified as not being highly effective cutting tools. Yet despite this, still no significant relationships were observed with manual variation. If task durations were extended relative to those recorded here, it is potentially the case that more substantial differences would have emerged between individuals of differing grip strength and manual anatomy. Previous experiments have, however, demonstrated similarly short cutting tasks to return significant relationships between tool performance and anatomy^27^, suggesting the lack of relationship identified here to be a meaningful signal. How these relationships interact with fatiguing across all stone tool types and during substantially longer cutting activities remains to be seen.

Participants were not instructed how to cut through the substrates–they used any cutting technique they deemed appropriate—but variation in cutting technique did appear to play a role in the likelihood of participants indicating they experienced fatiguing. Two main cutting techniques were employed: (1) bidirectional slicing and (2) unidirectional slicing. Bidirectional slicing was the most common method, and worked well for knives whose cutting edge was relatively long (i.e., over 60 mm). Unidirectional slicing consisted of quick, flick-of-the-wrist motions towards a participant’s body. Participants who utilised the latter method when using smaller knife forms displayed fewer fatigue-related comments and appeared to record quicker cutting times. Thus, we cannot rule out a role for differential cutting techniques influence the time records for these smaller knife forms. However, as these results were consistent with the larger knives, these differences do not appear to have impacted the main conclusion of this study. Rather, it highlights the potential impact that variation in cutting motions, with potential links to task familiarity and tool-use skill, can have on stone tool use experiments (e.g.^97^). This underlines the necessity of using statistically robust sample sizes and standardised experimental protocols for participants during stone tool-use experiments^98,99^. Finally, it is worth reemphasising that the morphology of all stone blades will be impacting the performance of tools (see Mika et al.^62^ for a detailed discussion), but the hierarchical Bayesian regression models used here took the different tool forms into consideration.

## Conclusion

Research investigating the evolution of the hominin hand has near exclusively focused on understanding when stone-tool related selective pressures started to influence manual anatomy, which tool behaviours created these evolutionary pressures, and how hominin anatomy may have responded. Little work has sought to understand how, when, why, or if these evolutionary pressures may have ended, or their impact been reduced, within our evolutionary history. Here we identified that the use of hafted technologies may have acted as a ‘technological equaliser’ between hominin individuals, reducing the impact that manual anatomical variation between individuals had on a tool’s performance. While no Palaeolithic populations would have exclusively used hafted knives and completely removed non-hafted flake technologies, it is potentially the case that specific anatomical advantages linked to precision gripping were no longer great enough to elicit evolutionary change. While we look forward to future experiments that retest these experimental results over more prolonged durations, for now at least, it appears that the advent of the handle (i.e., hafted stone technologies) may have marked the end of stone tool use behaviours creating a strong selective environment favouring more forceful precision gripping capabilities.

## Materials and methods

### Replica artefact manufacture

14 Clovis knife (point) forms were used in this study, with 30 of each being replicated on Texas Fredericksburg chert and hafted to a standardised handle (*n* = 420). For the first seven knife forms, we selected seven Clovis points representing the extreme bounds of known Clovis point shape space to replicate in stone and use in our experiments (see: Fig. 4^58^). Sites of interest were Simon (Knife #1), Shoop (Knives #2 and #5), Vail (Knife #3), Rummells-Maske (Knife #4), Anzick (Knife #6), and Bull Brook (Knife #7)^58^. These points are indicative of variation related to size, shape, and geographic regions^58^.

The remaining seven knives (i.e., Knives #8–14) were scaled versions of the first seven knife forms, and all have a consistent length of 79 mm (mm) (which is the average length of forms #1–7). Size was controlled to assess the impact of shape has on the functional performance of the knife types. The knife blades were produced by Craig Ratzat at Neolithics Flintknapping Supply House using modern lapidary equipment. He produced 30 ground specimens of each knife blade type, for a total *n* equalling 420. Ratzat, unaware of the goals of the experiment, then pressure flaked the edges of all knife blades to give them a sharp, archaeologically accurate edge. Across all fourteen knife blade types, point size ranged from 29.95 to 190.5 mm in length.

Modern knife handles typically range in diameter from 18.3 to 44.5 mm^100^, while Mastalerz et al.^101^ and Kong and Lowe^102^ have demonstrated ergonomically optimal handle diameters for adults to be between 20–30 mm or 25–30 mm (respectively), as they facilitate maximum gripping forces. Our handles fall within this optimal range and are comfortably located within the handle diameters seen in modern knives. Thus, all stone knives (points) were hafted to one-inch (25.4 mm) ash dowels shaped to fit the 14 knife forms (Supplementary Fig. S4). This was undertaken by Bob Berg at Thunderbird Atlatl, who dissolved kodak gelatine-based glue in warm water, and then used it with hemp fibre to haft the knives onto the dowels.

We note, as we have elsewhere, that we do not know exactly how Clovis points were hafted (p. 29)^56^ and encourage other cutting studies to explore the use of different hafting configuration and materials^61,62,103^.

### Knife measurements

Nine measurements were taken from each knife: (1) mass, (2) total length of the knife, (3) blade length, (4) handle length, (5) blade width, (6) handle width, (7) blade thickness, (8) handle thickness, and 9) hafting width. The locations of these measurements are displayed in Supplementary Fig. S5. Descriptive data for each of the knife groups can be found in Supplementary Table S4. Mass was recorded to the nearest gram (g), while the lengths, widths, and thicknesses were measured in millimetres (mm). Length and width measurements were taken using either a wooden ruler if the item was over 130 mm, or a digital calliper if under 130 mm. All knife measurements are available in the supplementary materials.

### Experimental procedure

**Figure 3:**
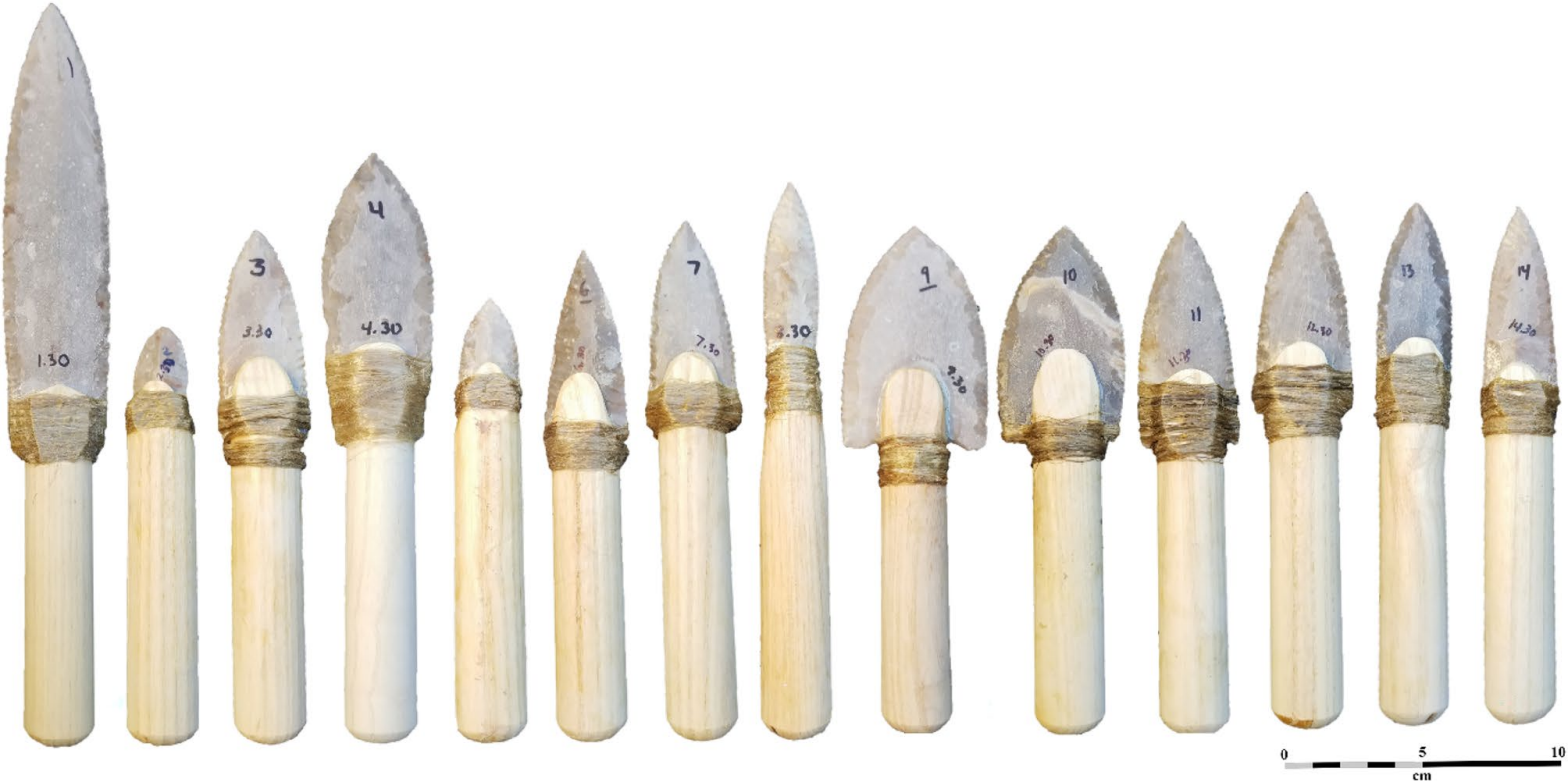
The fourteen hafted Clovis knife forms we used in the experiments.

30 participants (15 females, 15 males) were recruited to take part in the experiment from the student and staff population at Kent State University. Participants likely exhibited some variation in knife use experience, but any variation can be expected to be equally distributed through the participant sample. All can be assumed to have a fundamental familiarity with the use of metal knives during day-to-day activities. None had prior knowledge of the hypotheses under investigation. Each was presented with a set of 14 knives; one each from the 14 distinct Clovis forms described above (see Fig. 3). Each knife was subsequently engaged in two cutting tasks—one blade edge (side) was used for the first task, while the other was used for the second task (note that the blades were symmetrical along their long axis [i.e. perpendicular to the blade edge]). This mitigated for any role of blunting on tool performance. Knife forms were used in a randomly assigned order to control for potential fatiguing. Participation was volunteer-based, and all involved were unaware of the goals of the study. The age for participants ranged from 20 to 71 years (mean = 27.38, median = 24, SD = 10.57). Due to COVID protocols, all experiments took place outside.

In the first task, twisted sisal rope (0.25-in [~ 6 mm] diameter) was cut following previous studies that demonstrate its suitability for determining tool performance in precision-cutting tasks^27,36^. To secure the rope in place during cutting, two cast iron vices were bolted to two blocks of wood. The vices were 63.5 mm apart, allowing room for each tool to be moved and adjusted during use. Four, six-inch lengths of rope were secured into the left vice, before the ropes were twisted three times and clamped into the right vice. Participants were instructed to cut through the rope as quickly as possible, making sure there were no strands connected at the end of each trial. No instructions pertaining to cutting techniques were mentioned. The time taken (seconds) to cut all four rope lengths was recorded using a stopwatch and was one of our measures of cutting efficiency. The timer began at the first stroke and continued until the final length of rope was separated. The timer was paused when participants stopped to adjust their grip and continued once the cutting recommenced. We replaced the rope after each trial (i.e., the use of one knife), allowing the participants to take short breaks during the intervals.

The second cutting task required participants to cut through 103 red ceramic clay (C/06–2). Participants cut through blocks measuring approximately 42.5 × 40 × 41.25 mm (some minor variation was unavoidable between tasks). As with the rope task, we instructed participants to cut through the clay as quickly as possible, as though they were slicing through bread. The participants were timed in seconds, beginning with the initial slice. The timer stopped once the last slice completely disconnected the clay chunk from the block. In total, participants had to perform one cut with each knife (i.e., separate the block into two ‘sections’). Both the rope and clay tasks are intentionally very straightforward to help mitigate any skill-related differences between participants. No specialist knowledge is required to perform the task effectively, and the cutting action is fundamentally similar to many day-to-day tasks performed with modern metal tools.

There were some minor issues that occurred during testing (Supplementary Table S5). A few stone points fell out of their handles. This occurred ten times during Task 1—rope cutting; twice for Knife #1 (Simon), once for Knife #2 (Shoop #1), three times for Knife #5 (Shoop #2), three times for Knife #8, and once for Knife #10. This impacted Task 2—clay cutting—since the knives were no longer usable for cutting, resulting in incomplete trials. This occurred in Task 2—clay cutting as well: once for Knife #1 (Simon), and once for Knife #8. Participants commented on hearing cracking during knife use prior to blade detachment; upon examination of the joint, it was the glue that was the cause of the cracking sound. Another issue involved part of the blade edges snapping off during, or near, the final cut. The blade would hit either the wooden platform or the vice. In these cases, the blade was still usable for Task 2 since the opposite, non-damaged cutting edge was used for the second task. Throughout both tasks, this occurred three times for Knife #1 (Simon), once for Knife #2 (Shoop #1), once for Knife #5 (Shoop #2), and three times for Knife #13. In addition, the lashings were damaged once in Knife #8. All experimental data are available in the supplementary materials. All methods were performed in accordance with the relevant guidelines and regulations. The experiment was approved by the Kent State University Risk Mitigation sub-committee. Each trial was video recorded.

### Biometric variables

The biometric variables recorded from participants included hand length, lengths of digits one (1D), two (2D), and three (3D), grip strength (hook grip), and two pinch strengths (tip-to-tip and pad-to-side). Informed consent was obtained from all participants. Descriptive data for each of the biometric variables can be found in Table 3 (see also—Supplementary Fig. S6). Hand length was recorded from the top of 3D to the most proximal wrist line. Measurements for 1D to 3D were taken from the middle of the distal tip of the digit pulp, to the proximal line where the digit connects to the palm. All measurements of the hand were recorded in mm. Using these data, the ratio of 1D:2D was calculated. These variables have previously been recorded during earlier studies investigating links between tool-user biometric variation and stone tool use^22,27,28^.

**Table 3:** Descriptive statistics of the eight recorded biometric variables. Lengths were recorded in millimetres (mm). Strengths were recorded in kilograms (kg). The values displayed for the strengths are taken from the measurements from the user’s dominant hand.

Biometric Variable	Mean	SD	Minimum	Maximum	Range	Skewness
Age	27.23	10.57	20.00	71.00	51.00	3.32
Hand length	177.59	11.53	158.80	221.00	62.20	1.80
Thumb (D1) length	59.59	5.54	50.71	71.78	21.07	0.39
Index (D2) length	67.65	3.99	60.84	76.24	15.40	0.17
Middle (D3) length	74.06	5.20	64.00	87.15	23.15	0.04
1D:2D ratio	0.88	0.06	0.76	0.99	0.22	0.15
grip Strength	32.44	11.15	16.40	63.40	47.00	0.88
pad-to-side pinch strength	6.39	1.41	4.08	10.27	6.19	0.63
Tip-to-tip pinch strength	4.21	1.23	1.90	7.00	5.10	0.46

Grip strength was recorded using a Jamar Plus + Digital Hand Dynamometer. Grip and pinch strength were recorded in kilograms (kg). Three trials for each of the three strength measures were performed for both the left and right hand. The Jamar Plus + automatically calculated the average, standard deviation, and coefficient of variation for each hand. A number 2 handle position was used in most cases, but participants could adjust to handle position 3 if it increased comfort^104^. To perform the grip strength tests, participants began with their left hand, and were instructed to squeeze as hard as they could using a power grip. Once that number stabilized, the participant released their grip from the device. Upon completion of the first test participants repeated the test with their right hand using the same procedure. This alternating of hands continued until all six hand trials were completed. After the grip strength tests, participants were allotted a five-minute period of rest before moving onto the next set of strength tests.

**Figure 4:**
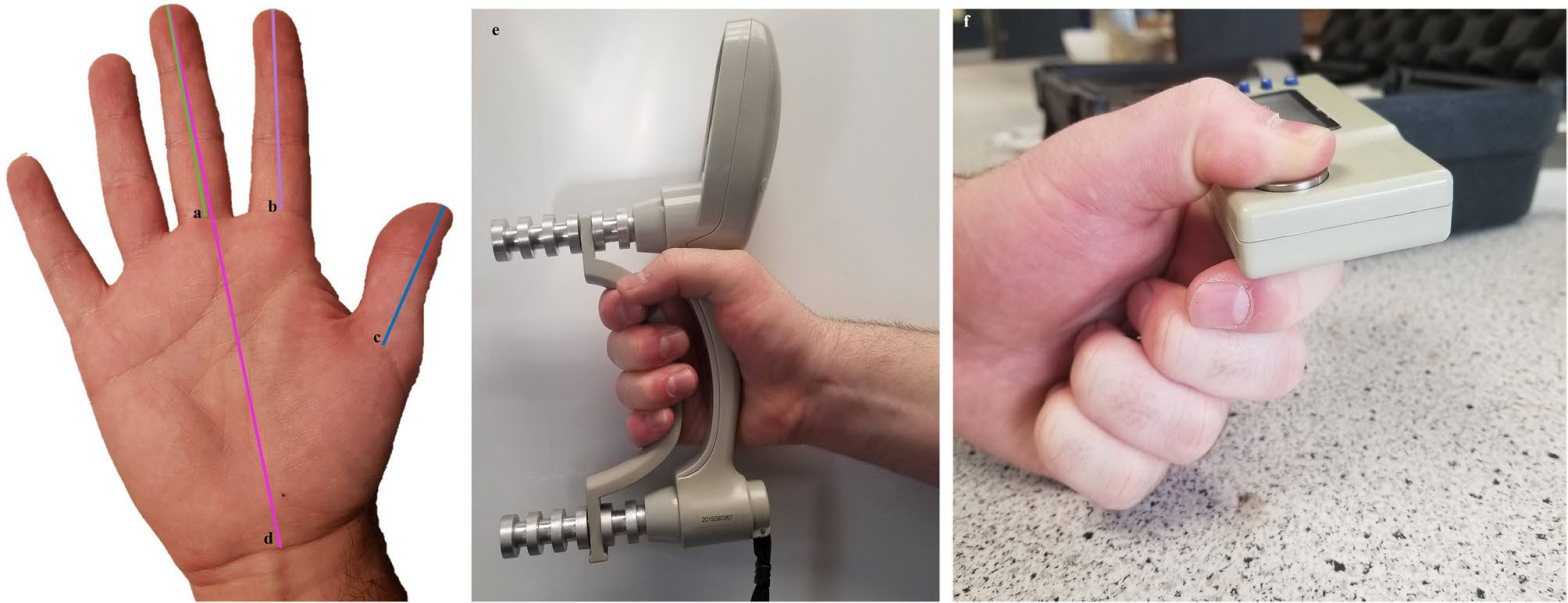
Example of where biometric variables were recorded: (**a**) middle (D3) digit; (**b**) index (D2) digit; (**c**) thumb (D1) digit; (**d**) hand measurement. c:b is 1D:2D ratio. (**e**) Example of grip strength in position 2, and (**f**) example of the pad-to-side pinch strength test, where the pad of the thumb was located on the superior metal plate, while the joint of the proximal and intermediate second phalanx rested on the inferior metal plate.

Pinch strengths were recorded using the Jamar Pinch Gauge—Plus + Digital—50 Lb. Capacity device. Beginning with the ‘Pad-to-Side’ Pinch Test, participants, using their dominant tool-using hand, placed their thumb (1D) on the superior metal plate and positioned the joint of their proximal and intermediate second phalanxes (2D) on the inferior metal plate, using digits 3D to 5D as buffers. Participants were then instructed to pinch as hard as they could until the numbers stabilized. This procedure was repeated for another two tests, and later averaged. After, the ‘Tip-to-Tip’ Pinch Strength was performed, each participant was instructed to place their first distal phalanx (1D) of their dominant tool-using hand on the superior metal plate and to place the distal phalanx of 2D on the inferior metal plate. 3D to 5D were not used to offer any support. Participants were instructed to pinch as hard as they could until the numbers stabilized. All values were recorded. Examples of where manual measurements were recorded, and how the grip and pinch strength tests were performed, can be found in Fig. 4.

### Stroke counts

The video records of each trial were used to record an additional record of tool use efficiency; the number of cutting strokes used. To do this, A.M., J.L., and A.S. watched the cutting trials at 0.25 × speed. Each reviewer used a click counter to record the number of knife strokes observed between a knife’s cutting edge first making contact with the worked substrate and it losing contact on the final cutting stroke. Stroke counts were averaged between the three analysts to generate the total number of counts per trial.

### Statistical analysis

We tested the influence of average pad-to-side pinch and grip strength of the dominant hand, hand and thumb length, and digit ratio on two different cutting tasks using a hierarchical Bayesian regression model implemented in R 4.2.2 (R Core Team) with the *brms* package. This approach was selected to adjust for the repeated measurements by all 30 subjects in the experiments with multiple knife types. The dependent variables for the cutting tasks are stroke count and time which are both approximately normally distributed. Both subject and knives are independent varying effects. Strength (either grip or pad-to-side pinch) is entered both as an independent variable and also allowed to vary as a random slope nested within knife type because different knives have different strength slopes. We set generic weakly informative priors (mean = 0, SD = 0.5) for all slopes and kept the *brms* default student t priors for the varying effects of individual and knife type. We used Gaussian distributions with identity linkages for the mean and variance. Sampling was carried out using the No-U-Turn Sampler (NUTS) developed by Hoffman and Gelman^105^. Final models were run with 2 chains for 10,000 iterations with a ‘warm-up’ of 5,000 iterations. The warm-up phase is used to determine the step size by maximizing the acceptance rate of proposals. For all parameters, r-hat values (a model diagnostic with expected value equal to 1) were exactly 1.00 and hence signify model convergence. Chains were also inspected visually for sufficient mixing to ensure that model results were appropriate. We used posterior distributions to make inferences about the strength of the effects in the model.

### Supplementary Information

Supplementary Information 1.

Supplementary Information 2.

## Data Availability

The data supporting this article are included in the supplementary material files.
