# Supplementary material for: Hafted technologies likely reduced stone tool-related selective pressures acting on the hominin hand
Source: Sci Rep. 2023 Sep 20;13:15582. doi: 10.1038/s41598-023-42096-z (PMC10511494; doi:10.1038/s41598-023-42096-z)
Supplement: Supplementary file 2 — Supplementary Information 2. [file 41598_2023_42096_MOESM2_ESM.pdf]

Supplementary Information for:

Hafted technologies likely reduced stone tool-related selective pressures acting on the hominin hand

Anna Mika [1, 2] *
Julie Lierenz [2, 3]
Andrew Smith [2]
Briggs Buchanan [4]
Robert S. Walker [5]
Metin I. Eren [2, 6]
Michelle R. Bebber [2]
Alastair Key [1]

[1] Department of Archaeology, University of Cambridge, Cambridge, CB2 3DZ, U.K.
[2] Department of Anthropology, Kent State University, Kent, Ohio, 44224, U.S.A.
[3] Department of Anthropology, Ohio State University, Columbus, Ohio, 43210, U.S.A.
[4] Department of Anthropology, University of Tulsa, Tulsa, Oklahoma, 74104, U.S.A.
[5] Department of Anthropology, University of Missouri, Columbia, 65211 U.S.A.
[6] Department of Archaeology, Cleveland Museum of Natural History, Cleveland, Ohio, 44106, U.S.A.

Supplementary Results

We carried out similar analyses as described in the main text on average pad-to-side pinch strength and grip strength of the dominant hand on two different cutting tasks using hierarchical Bayesian regression models. In this section we described additional analyses of the digit ratio, thumb length, and hand length.

Figures S1, S2, and S3 show that digit ratio, thumb length, and hand length generally have little overall relationship with the number of cut strokes or time for both tasks.

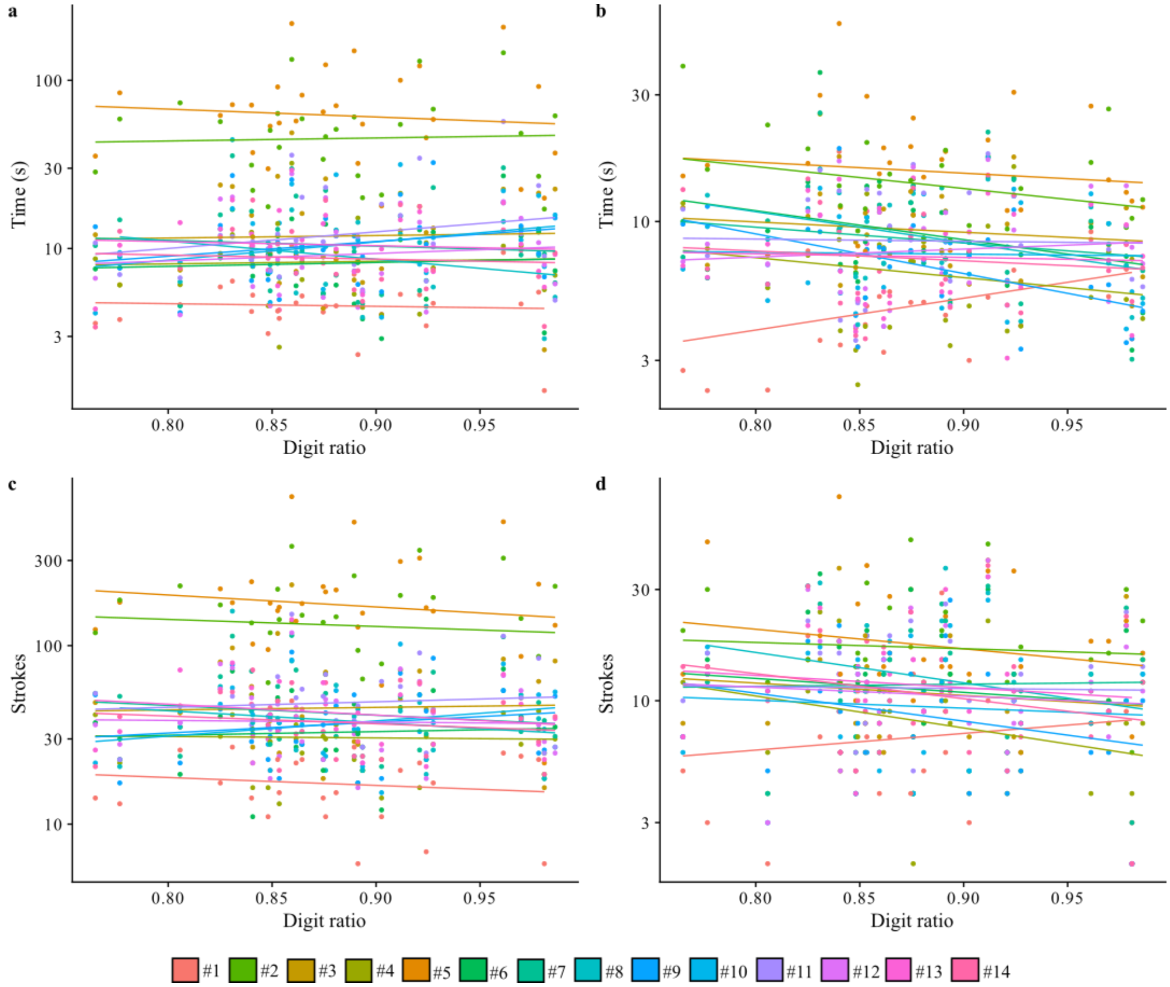

Supplementary Figure S1. Bivariate plots of digit ratio for the two tasks measured in time and number of strokes. Best-fit lines and individual observations are coloured by knife number. (**a**) digit ratio and time for task 1, (**b**) digit ratio and time for task 2, (**c**) digit ratio and stroke count for task 1, and (**d**) digit ratio and stroke count for task 2.

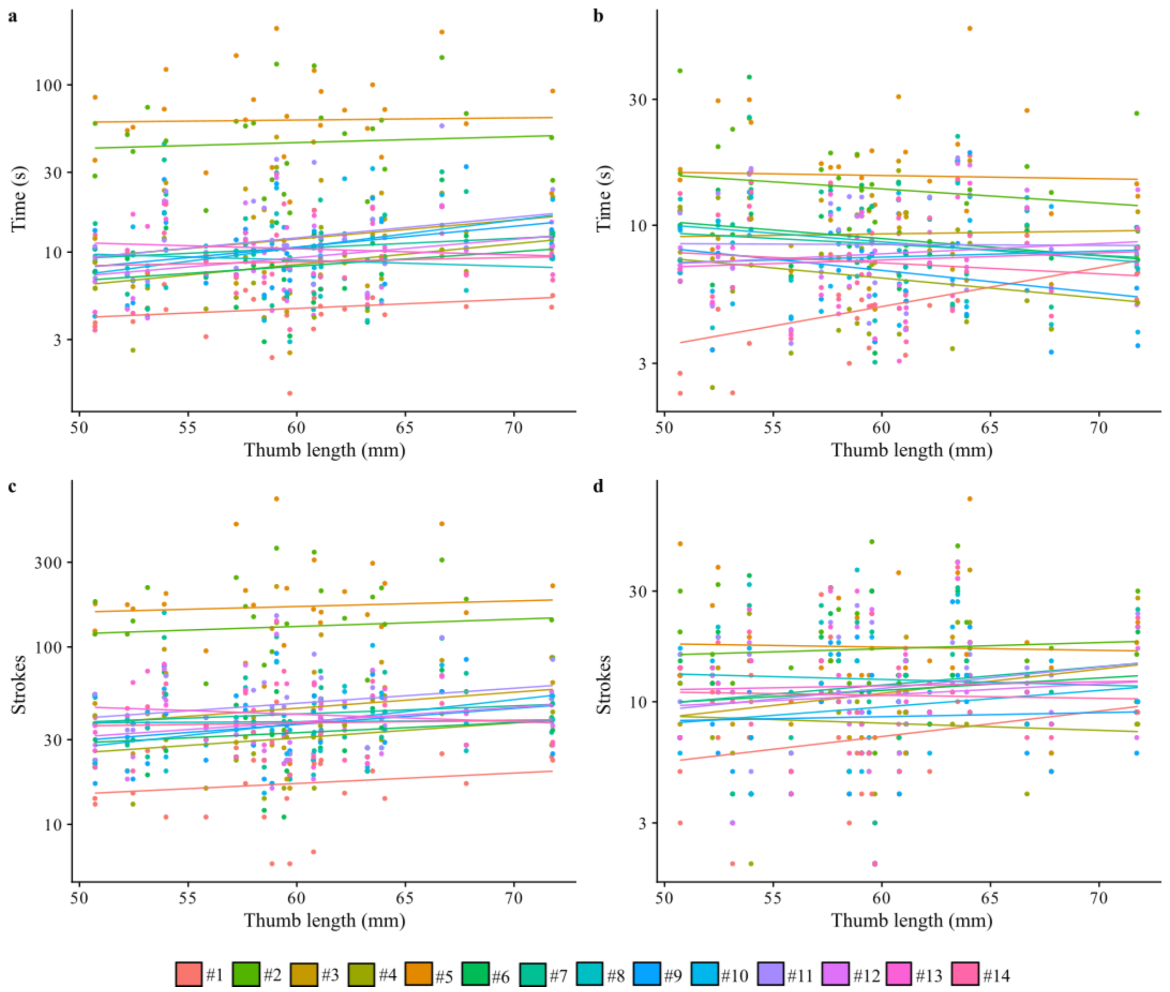

Supplementary Figure S2. Bivariate plots of thumb length for the two tasks measured in time and number of strokes. Best-fit lines and individual observations are coloured by knife number. (**a**) thumb length and time for task 1, (**b**) thumb length and time for task 2, (**c**) thumb length and stroke count for task 1, and (**d**) thumb length and stroke count for task 2.

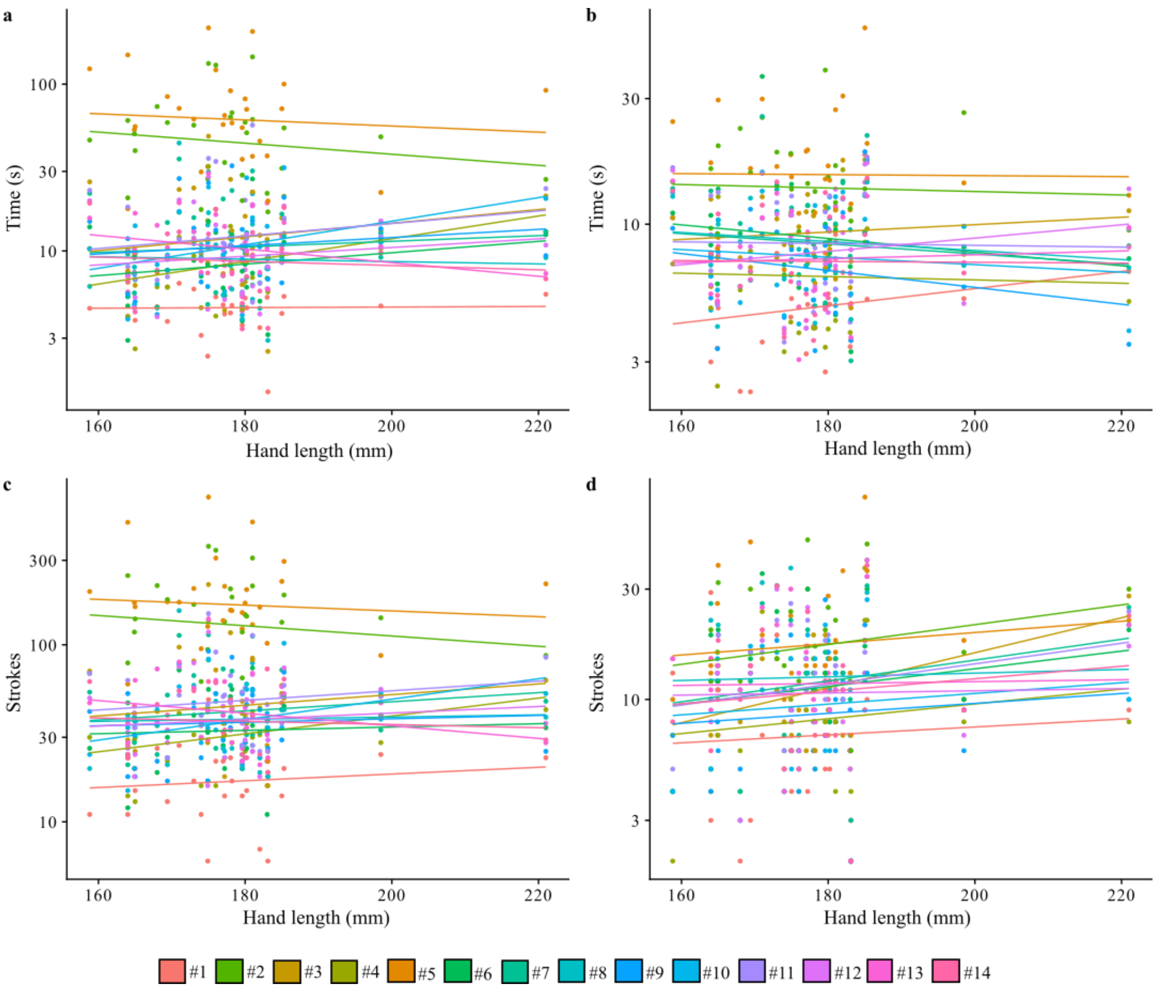

Supplementary Figure S3. Bivariate plots of hand length for the two tasks measured in time and number of strokes. Best-fit lines and individual observations are coloured by knife number. (**a**) hand length and time for task 1, (**b**) hand length and time for task 2, (**c**) hand length and stroke count for task 1, and (**d**) hand length and stroke count for task 2.

For all four models of digit ratio for the cutting tasks measured with stroke count or time the posterior distributions overlapped with zero suggesting that digit ratio is not an important factor (Table S1).

Supplementary Table S1. Bayesian regression model results for tests of digit ratio for stroke count and time for task 1 and 2. The lower (L-95% CI) and upper (U-95% CI) credible intervals for the posterior distribution of slopes all overlap zero indicating non-significance.

	**Estimate**	**Est. Error**	**L-95% CI**	**U-95% CI**
Model 1: Digit ratio, stroke count for task 1				
Intercept	0.13	12.84	29.85	79.87
Digit ratio	0.00	0.50	-0.99	0.99
Model 2: Digit ratio, stroke count for task 2				
Intercept	0.03	1.55	10.15	16.26

Digit ratio	0.00	0.50	-0.98	0.99
Model 3: Digit ratio, time for task 1				
Intercept	0.04	4.48	6.71	24.34
Digit ratio	0.00	0.50	-0.99	1.00
Model 4: Digit ratio, time for task 2				
Intercept	0.01	1.16	7.08	11.68
Digit ratio	0.00	0.50	-1.00	0.95

All four models of thumb length for the cutting tasks measured with stroke count or time the posterior distributions overlapped with zero suggesting that thumb length is not an important factor (Table S2).

Supplementary Table S2. Bayesian regression model results for tests of thumb length for stroke count and time for task 1 and 2. The lower (L-95% CI) and upper (U-95% CI) credible intervals for the posterior distribution of slopes all overlap zero indicating non-significance.

	**Estimate**	**Est. Error**	**L-95% CI**	**U-95% CI**
Model 1: Thumb length, stroke count for task 1				
Intercept	0.26	28.53	-10.54	102.29
Thumb length	0.00	0.44	-0.75	0.99
Model 2: Thumb length, stroke count for task 2				
Intercept	0.19	12.03	-14.48	32.97
Thumb length	0.00	0.20	-0.33	0.46
Model 3: Thumb length, time for task 1				
Intercept	0.19	15.95	-25.32	37.45
Thumb length	0.00	0.27	-0.39	0.68
Model 4: Thumb length, time for task 2				
Intercept	0.09	6.88	-0.98	26.12
Thumb length	0.00	0.11	-0.28	0.17

Similarly, all four models of hand length for the cutting tasks measured with stroke count or time the posterior distributions overlapped with zero suggesting that hand length is not an important factor (Table S3). As with pad-to-side pinch and grip strength, thumb length, and digit ratio, the estimates of the intercepts in these models shows that it takes on average more strokes and time to accomplish task 1, cutting through rope, than task 2, cutting through clay, when accounting for hand length.

Supplementary Table S3. Bayesian regression model results for tests of hand length for stroke count and time for task 1 and 2. The lower (L-95% CI) and upper (U-95% CI) credible intervals for the posterior distribution of slopes all overlap zero indicating non-significance.

	**Estimate**	**Est. Error**	**L-95% CI**	**U-95% CI**
Model 1: Hand length, stroke count for task 1				
Intercept	59.47	61.57	-61.07	180.95
Hand length	-0.03	0.34	-0.70	0.64
Model 2: Hand length, stroke count for task 2				
Intercept	-2.01	18.71	-38.39	35.27

Hand length	0.09	0.11	-0.12	0.29
Model 3: Hand length, time for task 1				
Intercept	18.83	26.30	-32.91	71.32
Hand length	-0.02	0.15	-0.31	0.27
Model 4: Hand length, time for task 2				
Intercept	11.44	10.08	-8.41	31.65
Hand length	-0.01	0.06	-0.12	0.10

Supplementary Figures and Tables

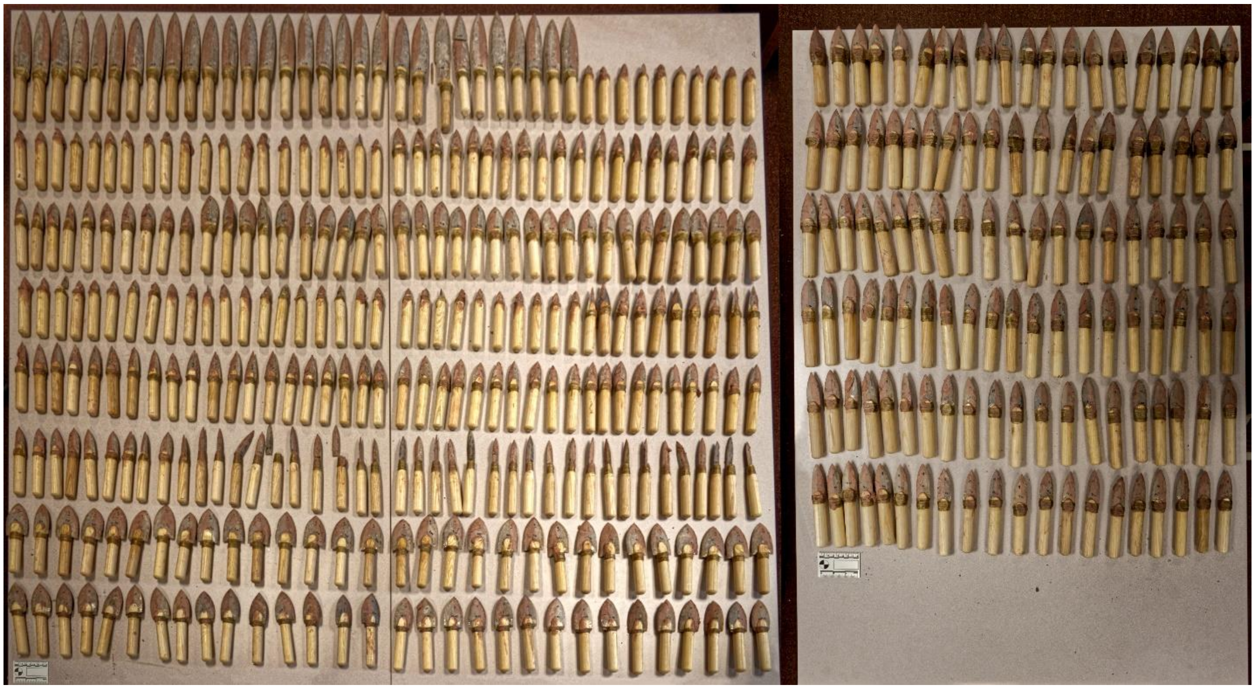

Supplementary Figure S4. The 420 hafted Clovis knife forms we used in the experiment.

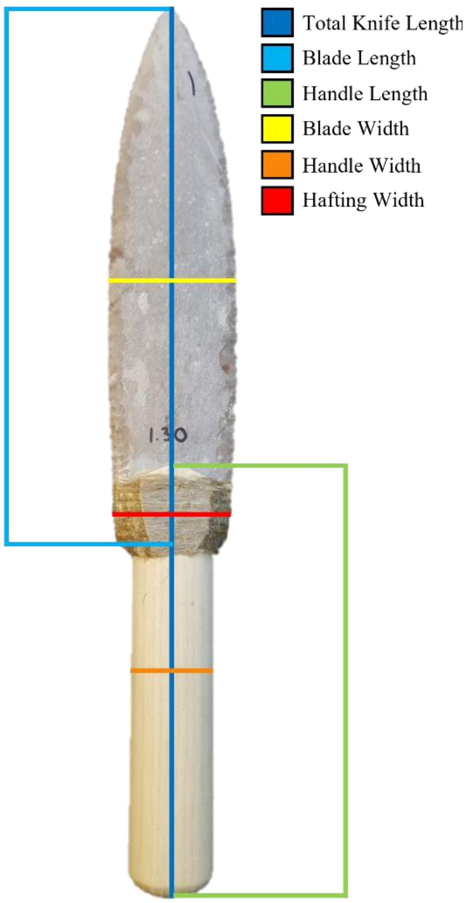

Supplementary Figure S5: The location of where knife measurements were recorded in millimetres (mm). Not shown: blade thickness and handle thickness.

Supplementary Table S4: Mean and standard deviation (SD) for each individual Clovis knife type. Mass was recorded in grams (g). Other measurements were recorded in millimetres (mm). Knife #9 does not have any recorded hafting widths due to the absence of hafting on the stone knife blade.

	**Mass**		**Total Knife Length**		**Blade Length**		**Handle Length**		**Blade Width**		**Handle Width**		**Blade Thickness**		**Handle Thickness**		**Hafting Width**	
**Knife Type**	*Mean*	*SD*	*Mean*	*SD*	*Mean*	*SD*	*Mean*	*SD*	*Mean*	*SD*	*Mean*	*SD*	*Mean*	*SD*	*Mean*	*SD*	*Mean*	*SD*
**#1**	110.67	7.18	278.68	4.99	184.03	4.25	126.16	1.32	39.10	1.17	23.95	0.61	7.81	0.68	24.01	0.60	34.60	2.03
**#2**	38.87	1.68	144.70	3.08	33.15	1.73	125.27	2.31	18.97	1.09	24.05	0.58	5.80	0.50	23.96	0.50	22.62	1.15
**#3**	47.20	3.10	168.07	4.01	70.48	1.44	123.77	2.62	27.58	1.14	23.75	1.09	6.49	0.59	23.41	0.58	30.71	1.14
**#4**	71.90	4.40	196.13	3.53	100.22	2.36	126.46	0.85	39.33	1.71	23.68	0.67	7.68	0.46	23.88	0.52	34.24	1.56
**#5**	41.13	1.17	146.36	2.17	32.73	1.42	125.17	1.53	18.11	0.84	24.01	0.49	5.41	0.59	24.13	0.56	21.26	1.00
**#6**	46.73	3.85	169.56	3.50	69.24	1.67	122.88	2.00	25.51	1.05	23.21	0.48	6.16	0.40	23.12	0.53	31.73	1.01
**#7**	47.43	3.07	169.07	4.04	67.78	1.30	123.20	1.68	26.74	0.97	23.71	0.75	6.37	0.47	23.30	0.65	29.32	1.18
**#8**	42.27	3.28	191.92	3.7	79.15	1.10	128.32	0.51	18.28	0.75	23.76	1.84	6.09	0.41	23.38	0.57	17.88	0.89
**#9**	68.17	5.50	178.86	4.21	82.63	1.78	125.33	2.61	47.02	2.10	23.40	0.57	7.37	0.91	23.43	0.62	-	-
**#10**	65.03	5.08	177.02	2.86	78.10	1.11	126.48	1.26	42.58	1.07	23.32	0.42	7.57	0.58	23.30	0.47	40.81	1.52
**#11**	52.83	3.74	173.86	3.63	78.16	1.54	126.18	1.88	31.53	1.07	23.73	0.73	6.57	0.56	23.39	0.69	36.00	1.05
**#12**	53.23	4.46	176.13	4.06	78.79	1.91	123.94	3.53	29.17	1.52	23.33	0.53	6.93	0.44	23.34	0.50	32.91	1.65
**#13**	51.30	4.62	181.73	3.76	79.31	1.30	126.41	1.76	27.52	0.77	23.59	0.55	7.28	0.57	23.46	0.57	26.91	0.76
**#14**	50.93	4.06	177.40	4.91	77.48	1.20	120.97	3.91	26.42	1.19	23.51	0.44	6.98	0.56	23.16	0.42	28.63	1.03

Supplementary Table S5: Knife breakage during testing for cutting tasks 1 and 2. "Broken" refers to specimens whose blades dislodged from their lashings and haft. "Damaged" refers to specimens whose knife blades were damaged with no impact on lashings. "Usable" refers to specimens whose blades were damaged but had intact lashings.

Knife Type	Number of specimens broken or damaged after task 1	Number of usable specimens (from the subset of the damaged or broken category) after task 1	Number of specimens broken or damaged after task 2	Number of usable specimens (from the subset of damaged or broken category) after task 2	Number or usable specimens (total survivors)	Total number of specimens broken or damaged (combined task 1 and 2)	Total percentage of specimens broken or damaged (combined task 1 and task 2)
#1	4	2	2	1	27	6	20.00%
#2	2	1	0	--	29	2	6.66%
#5	4	1	0	--	27	4	13.33%
#8	4	1	1	0	26	5	16.66%
#10	1	0	0	--	29	1	3.33%
#13	3	3	1	1	30	4	13.33%

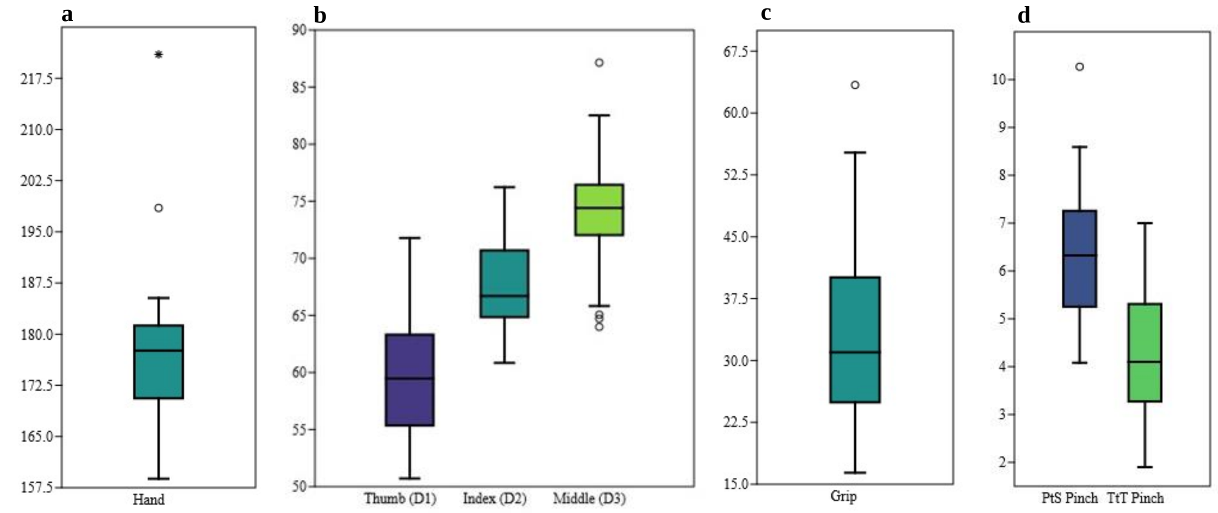

Supplementary Figure S6. Boxplots of participant data for (**a**) hand length (mm), (**b**) digit lengths for D1, D2, and D3 (mm), (**c**) grip strength (kg), and (**d**) pad-to-side (PtS) and tip-to-tip (TtT) pinch strengths (kg). Hand length, middle (d3) length, grip, and pts pinch strength have a few outliers.
